# Substance P induces fibrotic changes through activation of the RhoA/ROCK pathway in an in vitro human corneal fibrosis model

**DOI:** 10.1007/s00109-019-01827-4

**Published:** 2019-08-09

**Authors:** Marta Słoniecka, Patrik Danielson

**Affiliations:** 1 Department of Integrative Medical Biology, Umeå University, SE-901 87 Umeå, Sweden; 2 Department of Clinical Sciences, Ophthalmology, Umeå University, SE-901 87 Umeå, Sweden

**Keywords:** Cornea, Keratocytes, Scarring, Collagens, Fibrotic markers

## Abstract

Fibrosis is characterized by hardening, overgrowth, and development of scars in various tissues as a result of faulty reparative processes, diseases, or chronic inflammation. During the fibrotic process in the corneal stroma of the eye, the resident cells called keratocytes differentiate into myofibroblasts, specialized contractile fibroblastic cells that produce excessive amounts of disorganized extracellular matrix (ECM) and pro-fibrotic components such as alpha-smooth muscle actin (α-SMA) and fibronectin. This study aimed to elucidate the role of substance P (SP), a neuropeptide that has been shown to be involved in corneal wound healing, in ECM production and fibrotic markers expression in quiescent human keratocytes, and during the onset of fibrosis in corneal fibroblasts, in an in vitro human corneal fibrosis model. We report that SP induces keratocyte contraction and upregulates gene expression of collagens I, III, and V, and fibrotic markers: α-SMA and fibronectin, in keratocytes. Using our in vitro human corneal fibrosis model, we show that SP enhances gene expression and secretion of collagens I, III, and V, and lumican. Moreover, SP upregulates gene expression and secretion of α-SMA and fibronectin, and increases contractility of corneal fibroblasts during the onset of fibrosis. Activation of the preferred SP receptor, the neurokinin-1 receptor (NK-1R), is necessary for the SP-induced pro-fibrotic changes. In addition, SP induces the pro-fibrotic changes through activation of the RhoA/ROCK pathway. Taken together, we show that SP has a pro-fibrotic effect in both quiescent human keratocytes and during the onset of fibrosis in an in vitro human corneal fibrosis model.

**Electronic supplementary material:**

The online version of this article (10.1007/s00109-019-01827-4) contains supplementary material, which is available to authorized users.

## Introduction

The cornea is the transparent anterior part of the eye. In humans, it consists of five main layers: corneal epithelium, Bowman’s layer, corneal stroma, Descemet’s membrane, and corneal endothelium. The stroma is the thickest part of the cornea and comprises up to 90% of its thickness. The stroma is composed of collagen fibrils and cells called keratocytes [1, 2]. The main role of these cells is to maintain and secrete extracellular matrix (ECM) components such as collagens I, III, and V, lumican, and keratocan [3] in order to keep the cornea transparent during normal physiological conditions, but also during corneal injury and healing processes. Corneal injury triggers various reparative processes which will result in a normal stromal structure and function. However, when abnormal wound healing occurs, it might lead to corneal haze, scarring and fibrosis, and subsequent loss of vision [4]. One of the hallmarks of fibrosis is the generation of myofibroblasts from keratocytes [5]. This transition is promoted by transforming growth factor-beta (TGF-β) released by corneal epithelial cells [6]. Myofibroblasts are contractile fibroblast-like cells that express vimentin, alpha-smooth muscle actin (α-SMA), and desmin [4]. Moreover, they produce excessive amounts of ECM, including collagens I, III, and V, and fibronectin [7–9]. In addition to stimulation of α-SMA synthesis, TGF-β also upregulates expression of fibrillar collagen [10] and fibronectin [11]. The TGF-β induction of myofibroblast differentiation is driven by activation of Smad2/3/4 transcription factors; however, non-canonical pathways are also involved [12]. Ras homolog gene family member A (RhoA) is a small GTPase protein in the Rho family that is known to play a key role in such processes as cell adhesion, reorganization of actin cytoskeleton, cell migration, and differentiation of cells [13–15]. The Rho kinase (ROCK) family members are effectors of RhoA and have been shown to regulate actin polymerization [16] and trigger production of α-SMA and collagens in different types of fibroblasts [12]. Another non-canonical pathway involved in generation of myofibroblasts is activation of Ras-related C3 botulinum toxin substrate 1 (Rac1), another member of the small GTPase of the Rho family. It also plays a role in various processes, such as proliferation and migration, but it also has been shown to regulate cytoskeleton remodeling, cell adhesion, and motility [15, 17, 18]. In our previous study, we have shown that substance P (SP), an eleven amino acid long neuropeptide which belongs to the tachykinin family [19], triggers keratocyte migration during corneal wound healing through activation of these two small GTPases of the Rho family [15]. SP binds to neurokinin (NK-1, NK-2, and NK-3) receptors, with the highest affinity toward NK-1R [20, 21]. It is expressed in the central nervous system [22], and sensory neurons [23], where it is implicated in pain perception. However, it is also secreted by non-neuronal cells such as tumor cells [24], macrophages, neutrophils [25], tenocytes [26], corneal epithelial cells, and corneal keratocytes [27, 28]. Therefore, SP plays a role in diverse cellular and physiological processes such as immunomodulation [29], wound healing [30–32], and corneal epithelial cell migration [33, 34]. As mentioned earlier, our previous results showed that SP induces migration of keratocytes through activation of the Rac1/RhoA pathway and secretion of interleukin-8 (IL-8) [15]. Moreover, SP has been reported to be involved in modulating colitis-associated fibrosis [35], increasing liver fibrosis [36], and inducing fibrosis in cardiac fibroblasts [37]. Based on literature and our earlier results, we hypothesized that SP promotes fibrosis in the human corneal stroma through upregulation of fibrotic markers, such as fibronectin and α-SMA, and ECM components, such as collagens and lumican. Moreover, we wanted to study the mechanism behind the proposed SP pro-fibrotic actions. We aimed at testing the hypothesis in an in vitro human corneal fibrosis model.

## Materials and methods

### Collection of human corneas

Healthy human corneas kept in a corneal bank at the University Hospital of Umeå were used throughout this study. These corneas were obtained from deceased individuals who, when alive, chose to donate their corneas post-mortem for transplantation and research purposes, according to Swedish law (Table 1). If the corneas were not used for transplantation, either because they were not needed or because their expiration date for transplant was approaching, they were delivered to the laboratory. The study was vetted by the Regional Ethical Review Board in Umeå, which determined it to be exempt from the requirement for approval (2010-373-31M). The study was performed according to the principles of the Declaration of Helsinki.

**Table 1:** Corneas used throughout the study

Cornea	Age	Sex	Health status
1.	30	Male	Healthy
2.	60	Male	Healthy
3.	66	Male	Healthy
4.	66	Male	Healthy
5.	66	Male	Healthy
6.	66	Male	Healthy
7.	72	Male	Healthy
8.	75	Male	Healthy
9.	82	Male	Healthy
10.	84	Male	Healthy
11.	30	Female	Healthy
12.	91	Female	Healthy
13.	91	Female	Healthy

### Isolation of primary keratocytes

Isolation and culture of primary keratocytes were performed as previously described [28]. Briefly, remaining epithelial and endothelial cells were removed from the corneal surface with a scalpel. The central part of the cornea was cut out and cut into small pieces. The pieces were digested with 2 mg/ml collagenase (Sigma-Aldrich, # C0130) diluted in DMEM/F-12 + GlutaMAX™ medium (Thermo Fisher Scientific, Waltham, MA, USA, # 31330-095) containing 2% fetal bovine serum (FBS; Thermo Fisher Scientific, # 10082-147) and 1% penicillin-streptomycin (Thermo Fisher Scientific, # 15140-122) (DMEM/F-12 2% FBS), overnight at 37 °C. Samples were then centrifuged at 1500 rpm for 5 min. One part of the isolated cells was transferred into DMEM/F-12 2% FBS in order to maintain the proper phenotype of quiescent keratocytes. Remaining isolated cells were transferred to DMEM/F-12 + GlutaMAX™ medium containing 10% FBS and 1% penicillin-streptomycin (DMEM/F-12 10% FBS) in order to differentiate primary keratocytes into fibroblasts for further use in the in vitro fibrosis model. Cells were cultured at 37 °C with 5% CO_2_ until they reached confluency. Fresh medium (either DMEM/F-12 2% FBS or DMEM/F-12 10% FBS) was supplied every second or third day. 0.05% Trypsin-EDTA (Thermo Fisher, # 15400-054) was used to detach the cells, and the cells were split in a 1:2 or 1:3 ratio. Keratocytes and fibroblasts in passage 4 were used in this study. To assess the role of SP on the production of ECM by quiescent keratocytes, cells were grown and treated in DMEM/F-12 + GlutaMAX™ medium containing 0.1% FBS and 1% penicillin-streptomycin (DMEM/F-12 0.1% FBS). DMEM/F-12 10% FBS was used for the in vitro corneal fibrosis model and assessing the role of SP in the fibrosis process. The morphology of the cultured cells and the expression of keratocan is presented in Supplemental Figure 1. The corneas were assessed individually.

### In vitro human corneal fibrosis model

Corneal fibroblasts were plated on plastic culture dishes at desired densities (depending on the experimental method used; cf., each method in the “Materials and Methods” section for the exact cell density) in DMEM/F-12 10% FBS medium and stimulated by a stable vitamin C derivative L-Ascorbic acid 2-phosphate sesquimagnesium salt hydrate (VitC; Sigma-Aldrich, St. Louis, MO, USA, # A8960) at a concentration of 0.5 mM, and 0.25 ng/ml recombinant human TGF-β1 (R&D Systems, # 240-B) for up to 4 days. The concentration of both VitC and TGF-β1 were based on the fibrosis model developed by Karamichos et al. [38]. Fresh medium supplied with VitC and TGF-β1 was supplied every 2 days. The expression and secretion of ECM components and fibrotic markers collagen I, collagen III, collagen V, lumican, FN, and α-smooth muscle actin was assessed by western blot, RT-qPCR, ELISA, and flow cytometry 2 days and 4 days after stimulation. Gel contraction assay was used to assess the contractile abilities of newly formed myofibroblasts. Unstimulated fibroblasts in DMEM/F-12 10% FBS served as a control. To assess the role of SP in the onset of corneal fibrosis, corneal fibroblasts were additionally treated with SP (Sigma, St. Louis, MO, USA, # S6883) at a concentration of 10^−5^M [15]. In order to examine whether SP acts through activation of its preferred receptor, the neurokinin 1 receptor (NK-1R), cells were pre-treated with 10^−6^M L-733,060 [15] (Tocris, Bristol, UK, # 1145), a potent NK-1R antagonist, for 30 min at 37 °C. To study the mechanism of SP-induced changes, the ROCK (Rho-associated protein kinase) inhibitor Y-27632 (Merck, Kenilworth, NJ, USA, # SCM075), at a concentration of 10 μM, and Rac1 inhibitor NSC23766 (Tocris, # 2161), at a concentration of 50 μM, were used. The expression and secretion of ECM components and fibrotic markers: collagen I, collagen III, collagen V, lumican, fibronectin, EDA fibronectin (extra domain A fibronectin), and α-smooth muscle actin (α-SMA) was assessed by western blot, RT-qPCR, ELISA, and immunofluorescence 2 days and 4 days after stimulation. Gel contraction assay was used to assess the contractile abilities of newly formed myofibroblasts. Unstimulated fibroblasts in DMEM/F-12 10% FBS served as a control.

### RT-qPCR

Quiescent keratocytes were seeded into 6-well plates at a density of 0.25 × 10^6^ cells per well in DMEM/F-12 0.1% FBS 24 h before treatment. Keratocytes were treated with 10^−5^M SP for up to 8 days, with treatment repeated every 2 days. Corneal fibroblasts were seeded into 6-well plates at a density of 0.25 × 10^6^ cells per well in DMEM/F-12 10% FBS 24 h before treatment. Fibrosis was induced as described in the previous section. Desired wells were pre-treated with either 10^−6^M L-733,060 or Y-27632, and subsequently treated with 10^−5^M SP. Treatment was repeated every 2 days. Cells were lysed at day 2, day 4, and day 8 (keratocytes only), and mRNA was extracted using the RNA extraction kit (Qiagen, Venlo, Netherlands, # 74106) according to manufacturer’s instructions. Shortly, high-capacity cDNA reverse transcription kit (Thermo Fisher, # 4368813) was used to reverse transcribe 1000 ng of RNA into cDNA. Collagen I (COL1A1), collagen III (COL3A1), collagen V (COL5A1), lumican (LUM), fibronectin (FN), and α-smooth muscle actin (ACTA2) probes were used in order to determine gene expression (Thermo Fisher). Samples were run in duplicates in ViiA™ 7 Real-Time PCR System (Thermo Fisher). 18S and β-actin served as endogenous controls (Thermo Fisher; # 4333760F and # 4352935E, respectively). Analysis was performed with ViiA™ 7 Software (Thermo Fisher). Gene expression of the in vitro fibrosis samples was compared with gene expression of corneal fibroblasts in DMEM/F-12 10% FBS.

### Western blot

Corneal fibroblasts were seeded into 6-well plates at a density of 0.25 × 10^6^ cells per well in DMEM/F12 10% FBS 24 h before treatment. Fibrosis was induced as described earlier. Desired wells were pretreated with either 10^−6^ML-733,060 or Y-27632, and subsequently treated with 10^−5^M SP. Treatment was repeated every 2 days. Cells were freeze/thawed 3 times and further lysed with RIPA (radioimmunoprecipitation) lysis buffer (Thermo Fisher, # 89901) supplemented with protease and phosphatase inhibitor cocktail (Thermo Fisher, # 78446) 1 day, 2 days, and 4 days after treatment. Total protein concentration was assessed with Bradford assay (Bio-Rad, Hercules, CA, USA, # 5000006). SDS-polyacrylamide gels (Bio-Rad) were used to separate samples. Next, samples were transferred to PVDF membranes (GE Healthcare, Little Chalfont, UK, # GEHERPN303F) and blocked with 5% (*w*/*v*) bovine serum albumin (BSA; Sigma-Aldrich, # A9647) in TRIS-buffered saline (TBS) containing 0.1% Tween-20 (TBS-T; VWR, # 28829.183) for 1 h at room temperature and incubated overnight at 4 °C with rabbit polyclonal anti-alpha smooth muscle actin (α-SMA) antibody (Abcam, Cambridge, UK, # ab5694), rabbit monoclonal anti-RhoA antibody (Cell Signaling, Danvers, MA, USA, # 2117), or rabbit polyclonal anti-β-actin antibody (Cell Signaling, # 4967). Afterwards, membranes were washed in TBS-T and incubated with anti-rabbit IgG HRP-linked secondary antibody (Cell Signaling, # 7074) for 1 h at room temperature. Images were taken by the Odyssey® Fc imaging system (LI-COR, Lincoln, NE, USA). Additionally, densitometry analysis was performed using Image J analysis software (NIH).

### ELISA

0.3 × 10^4^ corneal fibroblasts were seeded into 96-well plates in DMEM/F-12 10% medium 24 h before treatment. Fibrosis was induced as described earlier. Desired wells were pretreated with either 10^−6^M L-733,060 or Y-27632, and subsequently treated with 10^−5^M SP. Treatment was repeated every 2 days. Supernatants were collected 2 days and 4 days after treatments. Secretion of pro-collagen I was assessed with Human Pro-Collagen I alpha 1 DuoSet ELISA (R&D Systems, #DY6220), collagen III with Human Collagen, type III, alpha 1 (COL3A1) ELISA kit (Cusabio, Houston, TX, USA, # CSB-E13446h), collagen V with Human Collagen, type V, alpha 1 (COL5A1) ELISA kit (Cusabio, # CSB-E13447h), lumican with Human Lumican DuoSet ELISA (R&D Systems, # DY2846), and fibronectin with Human Fibronectin DuoSet ELISA (R&D Systems, # DY1918), according to the manufacturer protocol.

### Immunofluorescence staining

10^4^ corneal fibroblasts were seeded in 8-well chamber slides (Corning, Corning, NY, USA # 354118). Desired wells were pretreated with Y-27632, and subsequently treated with 10^−5^M SP. Two days after treatment, cells were washed with phosphate-buffered saline (PBS) and fixed with 3.7% paraformaldehyde in 1× PBS for 20 min at room temperature. Next, cells were permeabilized with 0.1% Triton X-100 in 1× PBS for 5 min, and blocked with 1% BSA in PBS containing 0.1% Tween-20 (PBS-T) for 1 h. Afterwards, cells were incubated with mouse monoclonal anti-fibronectin (EDA) antibody for 1 h. Next, FITC-labeled secondary antibody (Dako, Glostrup, Denmark, #R0270) was incubated with the cells for 30 min. ProLong® Diamond Antifade Mountant with DAPI (Life Technologies, # P36962) was used to mount the cells. A Zeiss Axioskop 2 plus microscope equipped with epifluorescence and an Olympus DP70 digital camera were used for analysis.

10^4^ keratocytes (grown in DMEM/F-12 2% FBS) were seeded in 8-well chamber slides and supplemented with either DMEM/F-12 2% FBS or DMEM/F-12 0.1% FBS. Cells were incubated at 37 °C with 5% CO_2_ for 2 days. Cells were washed with phosphate-buffered saline (PBS) and fixed with 3.7% paraformaldehyde in 1× PBS for 5 min at room temperature. Next, cells were permeabilized with 0.1% Triton X-100 in 1× PBS for 5 min, and blocked with 1% BSA in PBS containing 0.1% Tween-20 (PBS-T) for 1 h. Afterwards, cells were incubated with mouse monoclonal anti-vimentin antibody (Abcam, ab8978), or rabbit polyclonal anti-keratocan antibody (Abcam, ab230175) at 4 °C, overnight. Next, FITC-labeled secondary antibody (Dako, Glostrup, Denmark, #R0270 and #F0205) was incubated with the cells for 1 h at room temperature. ProLong® Diamond Antifade Mountant with DAPI (Life Technologies, # P36962) was used to mount the cells. A Zeiss Axioskop 2 plus microscope equipped with epifluorescence and an Olympus DP70 digital camera were used for analysis.

### Contraction assay

Five hundred microliter (per well of a 24-well culture plate) of bovine collagen solution, type I PureCol® in DMEM/F-12 Medium, 5 mg/ml (0.5%) (Advanced BioMatrix, Carlsbad, CA, USA, # 5074-35ML) liquid gel was mixed together with 0.25 × 10^6^ keratocytes and 0.1% FBS. In order to assess the role of SP on the induction of contraction in quiescent keratocytes, the experimental group gels also contained 10^−5^M SP. Gels consisting only of keratocytes and 0.1% FBS served as controls. Gels were allowed to polymerize for 1 h at 37 °C. Next, gels were covered with DMEM/F-12 0.1% FBS with 10^−5^M SP in the experimental group. Afterwards, gels were detached from the wells and pictures were taken 0 h, 4 h, 1 day, 2 days, 3 days, and 4 days after treatments. Each treatment was performed in triplicates. Five hundred microliter (per well of a 24-well culture plate) of bovine collagen solution, type I PureCol® in DMEM/F-12 Medium, 5 mg/ml (0.5%) liquid gel was mixed together with 0.25 × 10^6^ corneal fibroblasts, FBS (10%), 0.5 mM VitC, and 0.25 ng/ml recombinant human TGF-β1. Experimental groups contained 10^−5^M SP, 10^−6^M L-733,060 with 10^−5^M SP, 10 μM Y-27632 with 10^−5^M SP, and 50 μM NSC23766 with 10^−5^M SP. Gels containing fibroblasts and 10% FBS only were also prepared and were used as negative controls for monitoring the progress of contraction in fibrosis induced gels. Gels containing fibroblasts, 10% FBS, VitC, and TGF-β1 served as positive controls for the fibrosis model and treated gels were compared with them. Gels were allowed to polymerize for 1 h at 37 °C. Next, gels were covered with DMEM/F-12 10% FBS containing 0.5 mM VitC, and 0.25 ng/ml recombinant human TGF-β1. Negative control gels were covered with DMEM/F-12 10% FBS only. 10^−5^M SP, 10^−6^M L-733,060 with 10^−5^M SP, 10 μM Y-27632 with 10^−5^M SP, and 50 μM NSC23766 with 10^−5^M SP were added to appropriate wells. Afterwards, gels were detached from the wells and pictures were taken 0 h, 4 h, 1 day, 2 days, 3 days, and 4 days after treatments. Each treatment was performed in triplicates. Photoshop (Adobe Systems, San Jose, CA, USA) was used to measure the area of the gels. Mean area was calculated for each experimental group at each time point. The results are shown as a percent of contraction: % contraction = (initial area − final area)/(initial area) × 100%. Representative images of contraction assays are shown in Supplemental Figure 2 and Supplemental Figure 3.

### Statistical analysis

All experiments were performed in triplicates. Data are presented as mean ± SD. Statistical analysis was performed with one-way ANOVA with Tukey’s post hoc test or unpaired *t* test. A *p* value of < 0.05 was considered statistically significant. Experiments were performed at least three times, meaning that at least three separate experiments were performed with cells isolated from different patients.

## Results

### SP induces contraction and upregulates gene expression of collagens and fibrotic markers in quiescent keratocytes

**Fig. 1:**
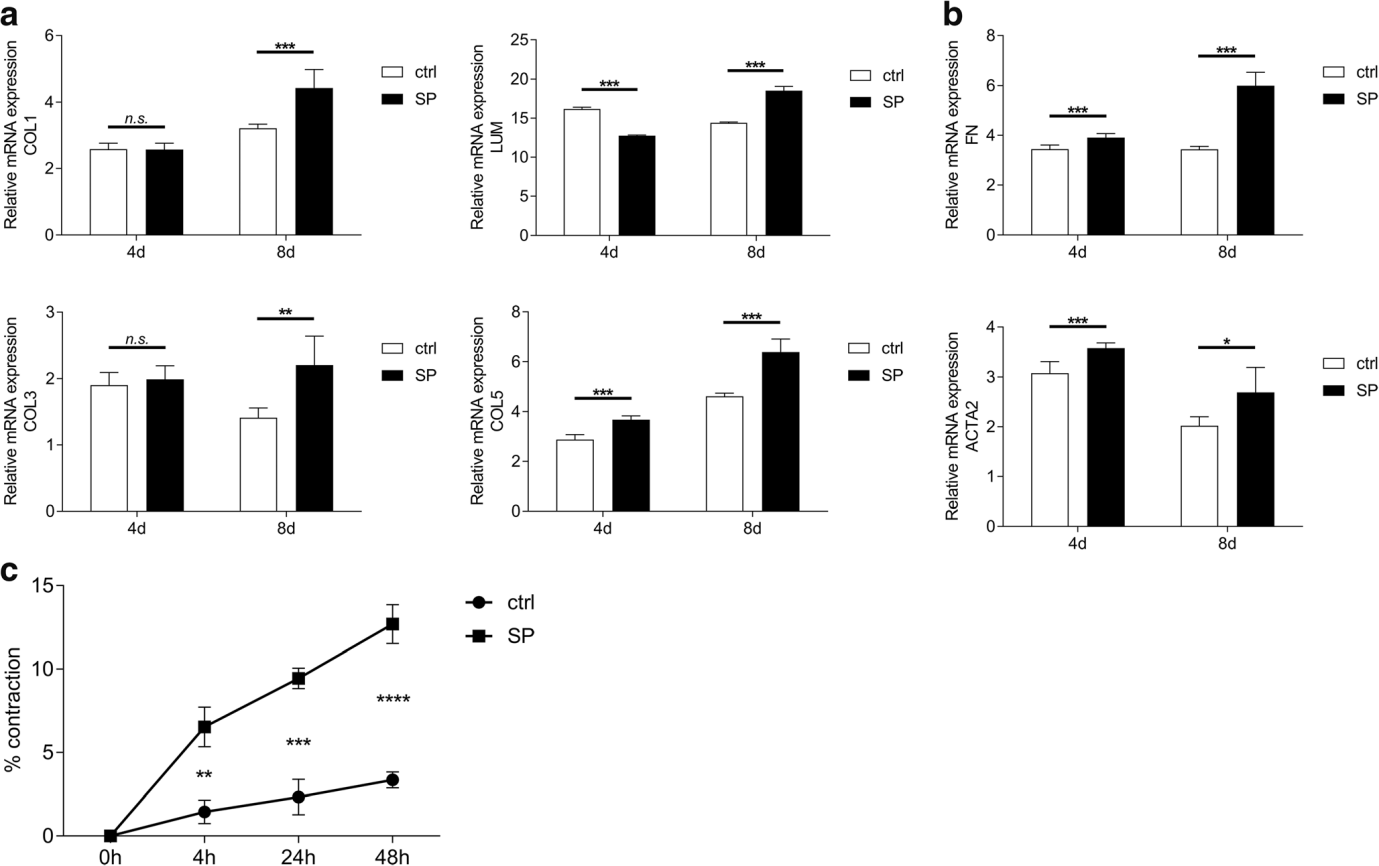
SP induces contraction and upregulates gene expression of collagens and fibrotic markers in quiescent keratocytes. **a** RT-qPCR showed that COL5A1 gene was upregulated both 4 days and 8 days after treatment with 10^−5^M SP. Gene expression of LUM was downregulated at day 4 but upregulated 8 days after the treatment. Gene expression of COL1A1 and COL3A1 was upregulated 8 days after treatment; however, it was unaffected 4 days after treatment. **b** RT-qPCR showed that gene expression of two fibrotic markers: FN and ACTA2 was upregulated both 4 days and 8 days after treatment with 10^−5^M SP. **c** Bovine collagen I liquid gel was mixed together with 0.25 × 10^6^ keratocytes and 0.1% FBS. The experimental group gels contained 10^−5^M SP. Gels consisting only of keratocytes and 0.1% FBS served as controls (ctrl). Gel contraction assay revealed that treatment of keratocytes with 10^−5^M SP results in increased contraction as soon as 4 h after treatment. Values are means ± SD. n.s. (not significant); *p < 0.05; **p < 0.01; ***p < 0.001; ****p < 0.0001

To test whether SP has an effect on gene expression of various ECM components, such as collagen I, collagen III, collagen V, and lumican, and on gene expression of fibrotic markers fibronectin and α-SMA, quiescent keratocytes were treated with 10^−5^M SP. Cells were lysed 4 days and 8 days after SP treatment and analyzed for COL1A1, COL3A1, COL5A1, LUM, FN, and ACTA2 gene expression. The results showed that COL5A1 gene was upregulated both 4 days and 8 days after treatment with 10^−5^M SP. However, expression of COL1A1 and COL3A1 was upregulated only 8 days after treatment, and it was unaffected 4 days after treatment. Interestingly, gene expression of LUM was downregulated at day 4 but upregulated 8 days after the treatment (Fig. 1a). Gene expression of FN and ACTA2 was upregulated 4 days and 8 days after treatment with 10^−5^M SP (Fig. 1b). Considering the gene expression results, we wanted to study whether SP would have an effect on keratocyte contraction, i.e., we wanted to test whether SP induces differentiation of keratocytes into fibroblasts and/or myofibroblasts. To test that, we used a gel contraction assay in which bovine collagen I liquid gel was mixed together with 0.25 × 10^6^ keratocytes and 0.1% FBS. In order to assess the role of SP on the induction of contraction in quiescent keratocytes, the experimental group gels contained 10^−5^M SP. Gels consisting only of keratocytes and 0.1% FBS served as controls. The results showed that treatment of keratocytes with 10^−5^M SP ended in contraction as soon as 4 h after treatment, suggesting that SP induces differentiation of keratocytes into fibroblasts and/or myofibroblasts (Fig. 1c).

### SP upregulates gene expression and protein secretion of various ECM components in corneal fibroblasts during the onset on fibrosis in vitro

**Fig. 2:**
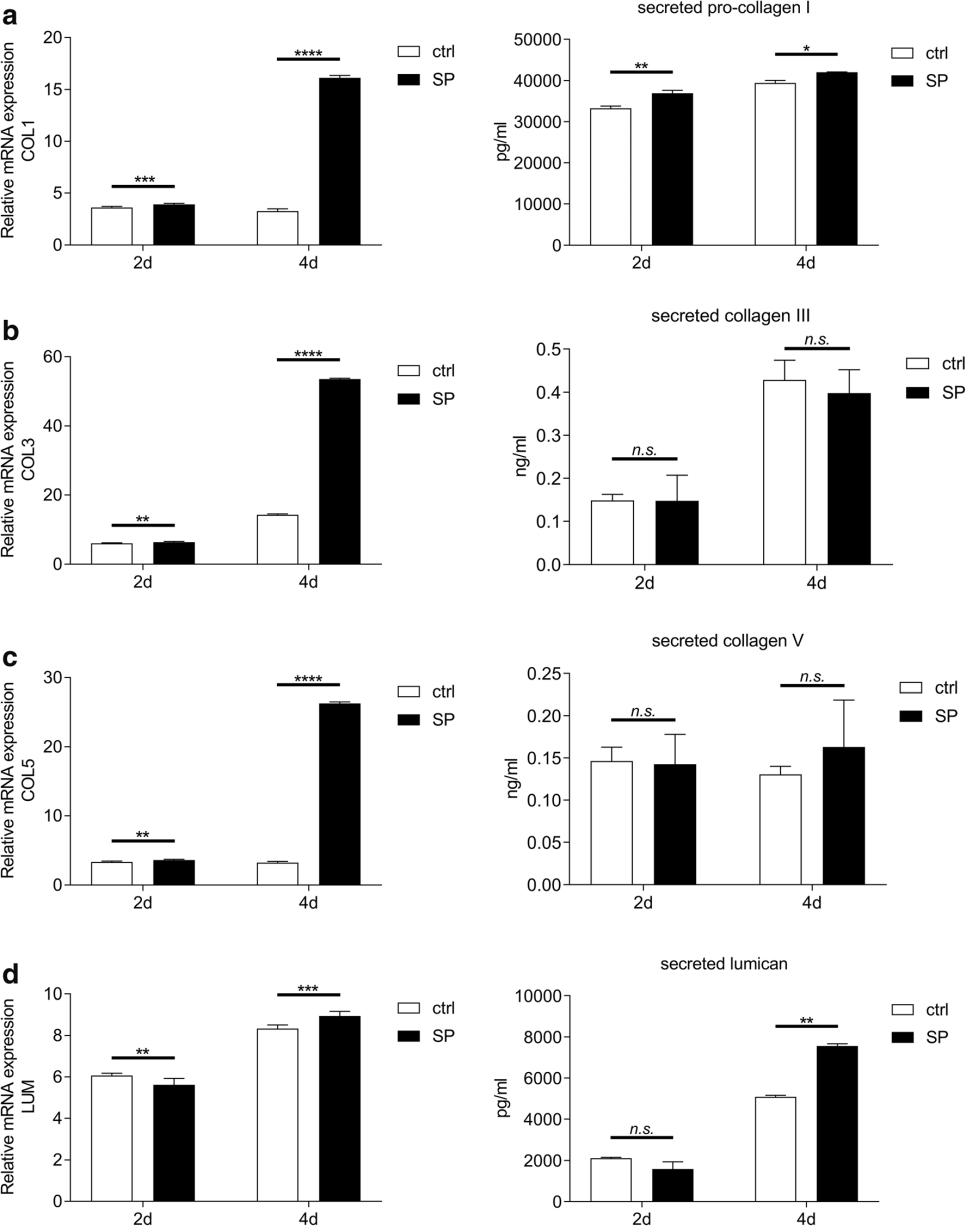
SP upregulates gene expression and protein secretion of various ECM components in corneal fibroblasts during the onset on fibrosis in vitro. **a** RT-qPCR showed that 10^−5^M SP upregulates COL1A1 gene expression in corneal fibroblasts both 2 days and 4 days after treatment and induction of fibrosis. Supernatants collected 2 days and 4 days after induction of fibrosis and treatment with 10^−5^M SP were subjected to pro-collagen I ELISA. Secretion of pro-collagen I was increased in supernatants collected from SP-treated cells both 2 days and 4 days after treatment. **b** 10^−5^M SP upregulated COL3A1 gene expression in corneal fibroblasts both 2 days and 4 days after treatment and induction of fibrosis. Supernatants collected 2 days and 4 days after induction of fibrosis and treatment with 10^−5^M SP were subjected to collagen III ELISA. SP treatment had no effect on collagen III secretion at either 2 days or 4 days after treatment. **c** 10^−5^M SP upregulated COL5A1 gene expression in corneal fibroblasts both 2 days and 4 days after treatment and induction of fibrosis. Supernatants collected 2 days and 4 days after induction of fibrosis and treatment with 10^−5^M SP were subjected to collagen V ELISA. SP treatment had no effect on collagen V secretion at either 2 days or 4 days after treatment. **d** 10^−5^M SP downregulated LUM gene expression in corneal fibroblasts at day 2, but 4 days after treatment and induction of fibrosis the LUM gene expression was increased. Supernatants collected 2 days and 4 days after induction of fibrosis and treatment with 10^−5^M SP were subjected to lumican ELISA. SP treatment had no effect on lumican secretion at day 2; however, it increased lumican secretion 4 days after treatment. Values are means ± SD. n.s. (not significant); *p < 0.05; **p < 0.01; ***p < 0.001; ****p < 0.0001

To study the possible role of SP in the onset of fibrosis in the cornea, we used an in vitro human corneal fibrosis model. In this model, corneal fibroblasts are stimulated with 0.5 mM VitC and 0.25 ng/ml recombinant human TGF-β1 in DMEM/F-12 10% FBS medium for up to 4 days. First, we sought to study whether SP affects gene expression and secretion of collagen I, collagen III, collagen V, and lumican. To test that, fibrosis was induced in corneal fibroblasts and the cells were subsequently treated with 10^−5^M SP. Supernatants were collected 2 and 4 days after treatment, and were subjected to collagen I, collagen III, collagen V, and lumican ELISA. Additionally, cells were lysed 2 days and 4 days after SP treatment and analyzed for COL1A1, COL3A1, COL5A1, and LUM gene expression. The results showed that SP upregulates COL1A1 gene expression in corneal fibroblasts both 2 days and 4 days after treatment. Secretion of pro-collagen I was increased in supernatants collected from SP-treated cells both 2 days and 4 days after treatment (Fig. 2a). COL3A1 expression was also upregulated both 2 days and 4 days after treatment. However, SP treatment had no effect on collagen III secretion at neither 2 days nor 4 days after treatment (Fig. 2b). COL5A1 expression was upregulated both 2 days and 4 days after treatment. However, SP treatment had no effect on collagen V secretion at neither 2 days nor 4 days after treatment (Fig. 2c). Expression of LUM was decreased by SP at day 2, but it increased 4 days after treatment. SP treatment had no effect on lumican secretion at day 2; however, it increased lumican secretion 4 days after treatment (Fig. 2d).

### SP increases contractile abilities, and upregulates gene expression, protein secretion, and expression of fibrotic markers fibronectin and α-SMA in corneal fibroblasts during the onset on fibrosis in vitro

**Fig. 3:**
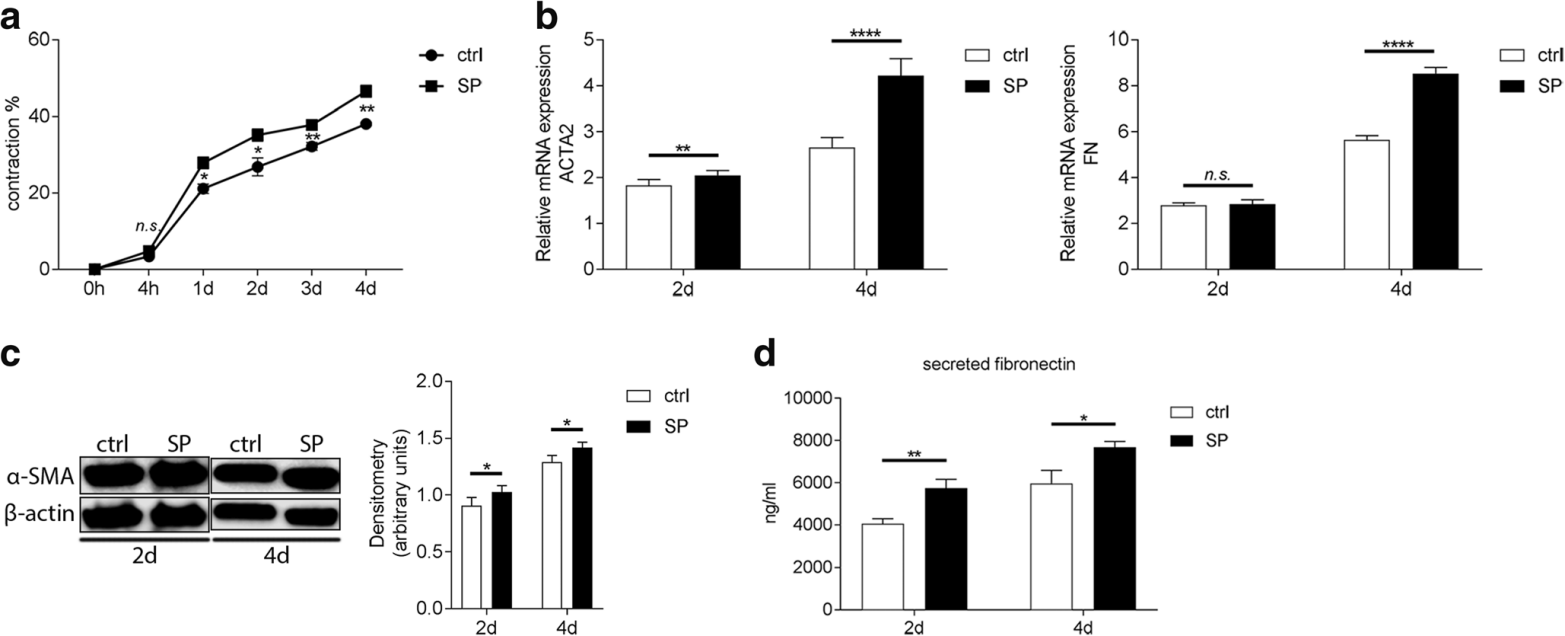
SP increases contractile abilities, and upregulates gene expression, protein secretion, and expression of fibrotic markers fibronectin and α-SMA in corneal fibroblasts during the onset on fibrosis in vitro. **a** Bovine collagen I liquid gel was mixed together with 0.25 × 10^6^ corneal fibroblasts, FBS (10%), 0.5 mM VitC, and 0.25 ng/ml recombinant human TGF-β1. Experimental groups were treated with 10^−5^M SP. Gels containing fibroblasts, 10% FBS, VitC, and TGF-β1 served as positive controls for the fibrosis model (ctrl) and treated gels were compared with them. Gel contraction assay showed that 10^−5^M SP increased contractile abilities of corneal fibroblasts already at day 1 when compared with positive control. **b** RT-qPCR showed that 10^−5^M SP upregulated ACTA2 gene expression in corneal fibroblasts both 2 days and 4 days after treatment and induction of fibrosis. Gene expression of FN was unaffected by SP treatment at day 2; however, it was significantly increased 4 days after treatment. **c** α-SMA protein expression was assessed by western blot at 2 days and 4 days after treatment with 10^−5^M SP and induction of fibrosis. Densitometry analysis was performed in order to quantify the western blot results. Intensity of α-SMA band was normalized to the intensity of β-actin band. SP treatment resulted in increased α-SMA (42 kDa) protein expression both 2 days and 4 days after treatment. β-actin (45 kDa) served as loading control. **d** Supernatants collected 2 days and 4 days after induction of fibrosis and treatment with 10^−5^M SP were subjected to fibronectin ELISA. Secretion of fibronectin was increased in supernatants collected from SP-treated cells both 2 days and 4 days after treatment. Values are means ± SD. n.s. (not significant); *p < 0.05; **p < 0.01; ****p < 0.0001

The next step in assessing the possible role of SP in the onset of corneal fibrosis was to determine whether SP affects gene and protein expression of two fibrotic markers: α-SMA and fibronectin. First, we assessed whether SP induced differentiation of fibroblasts into myofibroblasts, which are a hallmark of fibrosis. In order to study this, we used a gel contraction assay in which bovine collagen I liquid gel was mixed together with 0.25 × 10^6^ corneal fibroblasts, FBS (10%), 0.5 mM VitC, and 0.25 ng/ml recombinant human TGF-β1. Experimental groups contained 10^−5^M SP. Gels containing fibroblasts, 10% FBS, VitC, and TGF-β1 served as positive controls for the fibrosis model and the SP-treated gels were compared with them. The results showed that SP increases contractile abilities of corneal fibroblasts already at day 1 when compared with positive control. This effect was observed throughout the duration of the experiment (Fig. 3a). Next, we wanted to test whether the SP-increased contractile abilities of fibroblasts correlate with gene expression of ACTA2 and FN. RT-qPCR showed that 10^−5^M SP upregulates ACTA2 gene expression in corneal fibroblasts both 2 days and 4 days after treatment. Gene expression of FN was unaffected by SP treatment at day 2; however, it was significantly increased 4 days after treatment (Fig. 3b). Moreover, SP increased expression of α-SMA protein in treated fibroblasts both 2 days and 4 days after treatment as assessed by western blot (Fig. 3c). Secreted (extracellular) fibronectin levels were increased in supernatants collected from SP-treated cells both 2 days and 4 days after treatment (Fig. 3d).

### SP induces pro-fibrotic changes in corneal fibroblasts through activation of its receptor NK-1R

**Fig. 4:**
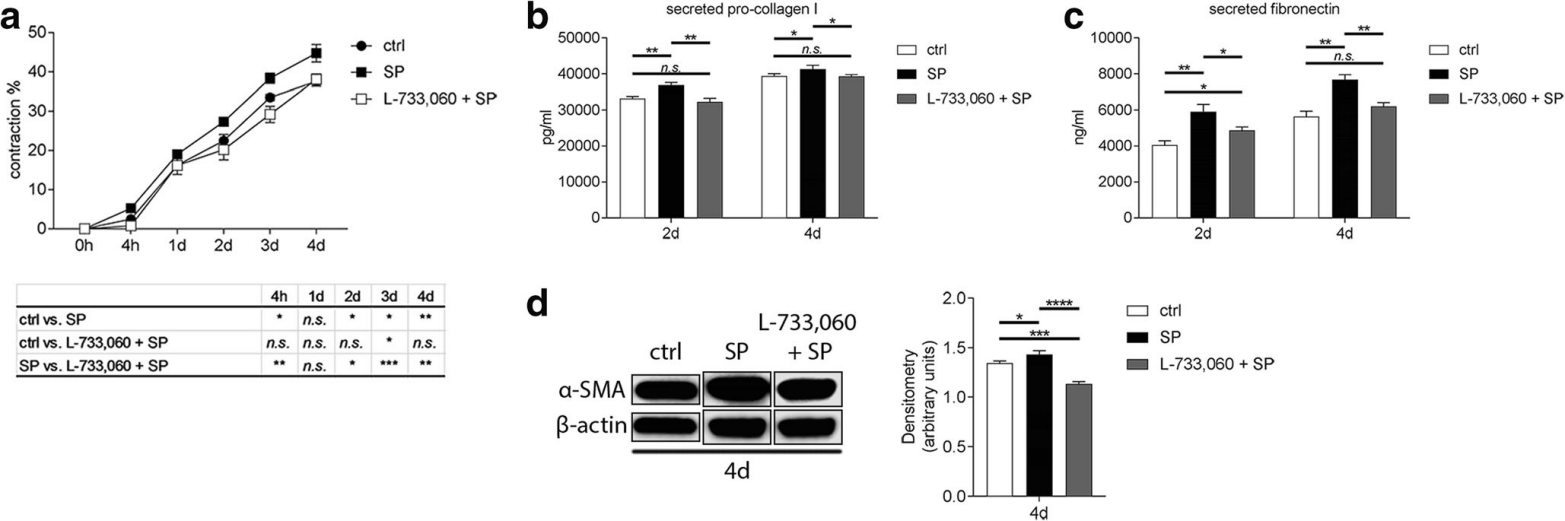
SP induces pro-fibrotic changes in corneal fibroblasts through activation of its receptor NK-1R. **a** Bovine collagen I liquid gel was mixed together with 0.25 × 10^6^ corneal fibroblasts, FBS (10%), 0.5 mM VitC, and 0.25 ng/ml recombinant human TGF-β1. Experimental groups contained 10^−5^M SP or 10^−6^M of the NK-1R blocker L-733,060 together with 10^−5^M SP. Gels containing fibroblasts, 10% FBS, VitC, and TGF-β1 served as positive controls for the fibrosis model (ctrl) and treated gels were compared with them. Gel contraction assay showed that when NK-1R receptor was blocked with L-733,060, SP was unable to induce contraction of the gels. Gel contraction of L-733,060 treated gels remained at the level of control. **b** Supernatants collected 2 days and 4 days after induction of fibrosis and treatment with 10^−5^M SP or 10^−6^M L-733,060 together with 10^−5^M SP were subjected to pro-collagen I ELISA. Secretion of pro-collagen I was increased in supernatants collected from SP-treated cells both 2 days and 4 days after treatment. Treatment of cells with L-733,060 and SP together resulted in decreased secretion of pro-collagen I as compared with SP alone. **c** Supernatants collected 2 days and 4 days after induction of fibrosis and treatment with 10^−5^M SP or 10^−6^M L-733,060 together with 10^−5^M SP were subjected to fibronectin ELISA. Secretion of fibronectin was increased in supernatants collected from SP-treated cells both 2 days and 4 days after treatment. Treatment of cells with L-733,060 and SP together resulted in decreased secretion of fibronectin as compared with SP alone. **d** α-SMA protein expression was assessed by western blot 4 days after treatment with 10^−5^M SP or 10^−6^M L-733,060 together with 10^−5^M SP. Densitometry analysis was performed in order to quantify the western blot results. Intensity of α-SMA band was normalized to the intensity of β-actin band. SP treatment resulted in increased α-SMA (42 kDa) protein expression 4 days after treatment. Treatment of cells with L-733,060 and SP together resulted in decreased expression of α-SMA as compared with SP alone. β-actin (45 kDa) served as loading control. Values are means ± SD. n.s. (not significant); *p < 0.05; **p < 0.01; ***p < 0.001; ****p < 0.0001

Next, we wanted to evaluate if SP induces pro-fibrotic changes through activation of its preferred receptor, NK-1R. First, we assessed whether the activation of the NK-1R is needed for the SP-induced contraction of the collagen gels, in which bovine collagen I liquid gel was mixed together with 0.25 × 10^6^ corneal fibroblasts, FBS (10%), 0.5 mM VitC, and 0.25 ng/ml recombinant human TGF-β1. Experimental groups were treated with 10^−5^M SP or 10^−6^M L-733,060 with 10^−5^M SP. Gels containing fibroblasts, 10% FBS, VitC, and TGF-β1 served as positive controls for the fibrosis model and treated gels were compared with them. The results showed that when the NK-1R is blocked with the inhibitor, SP was unable to increase contraction of the gels, and the gel contraction remained at the level of control (Fig. 4a). Next, we wanted to study if blocking the NK-1R receptor will affect secretion of pro-collagen I, fibronectin, and expression of α-SMA. Treatment of cells with L-733,060 together with 10^−5^M SP resulted in decreased secretion of pro-collagen I when compared with SP treatment only (Fig. 4b). The same effect was observed for fibronectin secretion (Fig. 4c). Western blot analysis revealed that inhibition of the NK-1R receptor results in decreased expression of α-SMA when compared with SP treatment only (Fig. 4d).

### SP induces pro-fibrotic changes in corneal fibroblast by activation of the RhoA/ROCK pathway

**Fig. 5:**
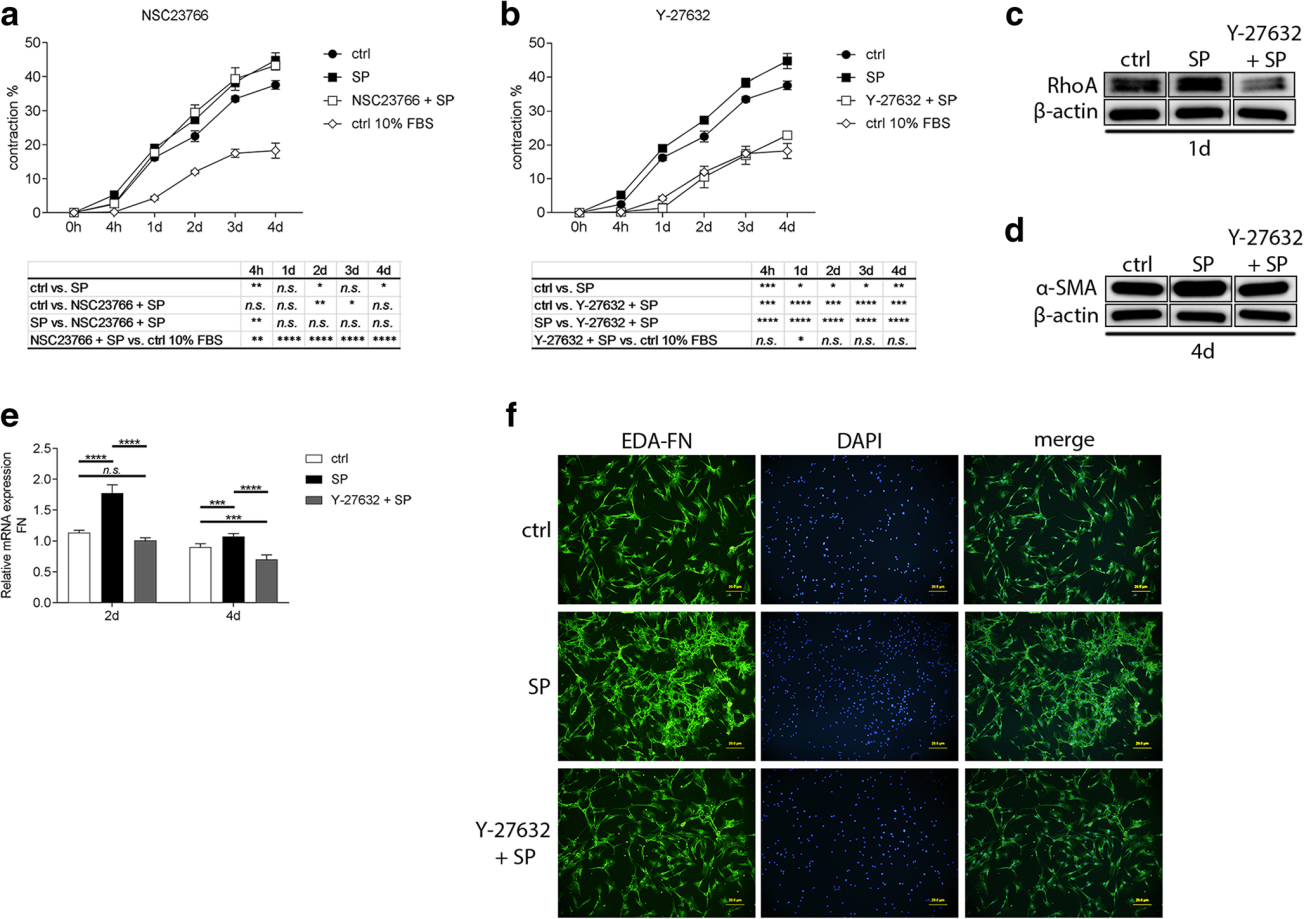
SP induces pro-fibrotic changes in corneal fibroblasts by activation of the RhoA/ROCK pathway. Five hundred microliter (per well of a 24-well culture plate) of bovine collagen solution, type I PureCol® in DMEM/F-12 Medium, 5 mg/ml (0.5%) liquid gel was mixed together with 0.25 × 10^6^ corneal fibroblasts, FBS (10%), 0.5 mM VitC, and 0.25 ng/ml recombinant human TGF-β1. Experimental groups contained 10^−5^M SP, 50 μM NSC23766 (Rac1 inhibitor) together with 10^−5^M SP, or 10 μM Y-27632 (ROCK inhibitor) together with 10^−5^M SP. Gels containing fibroblasts, 10% FBS, VitC, and TGF-β1 served as positive controls for the fibrosis model (ctrl) and treated gels were compared with them. **a** Gel contraction assay showed that when the Rac1 pathway was blocked with NSC23766, SP was still able to increase contraction of the gels as much as SP alone. **b** Gel contraction assay showed that when the RhoA/ROCK pathway was blocked with Y-27632, SP was unable to increase contraction of the gels. In fact, the gel contraction remained on the level of the negative control (ctrl 10% FBS; corneal fibroblasts and 10% FBS only gels), abolishing also the fibrosis model effect. **c** Western blot analysis of RhoA expression in corneal fibroblast stimulated with 10^−5^M SP or treated with 10 μM Y-27632 + 10^−5^M SP. SP stimulation resulted in increased expression of RhoA (21 kDa) 1 day after treatment. Y-27632 treatment resulted in decreased expression of RhoA. β-actin (45 kDa) served as loading control. **d** Western blot analysis of α-SMA expression in corneal fibroblast stimulated with 10^−5^M SP or treated with 10 μM Y-27632 + 10^−5^M SP. SP stimulation resulted in increased expression of α-SMA (42 kDa) 4 days after treatment. Y-27632 treatment resulted in decreased expression of α-SMA. β-actin (45 kDa) served as loading control. **e** RT-qPCR showed that 10^−5^M SP upregulated FN gene expression in corneal fibroblasts both 2 days and 4 days after treatment and induction of fibrosis. Treatment of cells with Y-27632 inhibitor resulted in decreased FN expression at both 2 days and 4 days. **f** 10^4^ corneal fibroblasts were seeded in an 8-well chamber slides. Desired wells were pretreated with 10 μM Y-27632, and subsequently treated with 10^−5^M SP. Two days after treatment, cells were fixed, permeabilized, and incubated with mouse monoclonal anti-fibronectin (EDA-FN) antibody for 1 h. SP treatment increased expression of EDA-FN (FITC, green). Treatment of cells with Y-27632 inhibitor together with 10^−5^M SP showed that expression of fibronectin (EDA) was decreased. DAPI (blue) was used to visualize nuclei. Values are means ± SD. n.s. (not significant); *p < 0.05; **p < 0.01; ***p < 0.001; ****p < 0.0001

As activation of the Rac1 and RhoA pathways has been implicated in promoting fibrosis, and as we have shown in our previous studies that SP activates both of these pathways [15], we sought to study whether the SP-induced pro-fibrotic changes are due to activation of either of the Rac1 or RhoA pathways. We used Rac1 inhibitor NSC23766 and RhoA/Rho kinase (ROCK) inhibitor Y-27632. We further used gel contraction assay to assess the involvement of the pathways in the SP-induced contraction. The bovine collagen I liquid gel was mixed together with 0.25 × 10^6^ corneal fibroblasts, FBS (10%), 0.5 mM VitC, and 0.25 ng/ml recombinant human TGF-β1. Experimental groups contained 10^−5^M SP, 50 μM NSC23766 with 10^−5^M SP, and 10 μM Y-27632 with 10^−5^M SP. Gels containing fibroblasts, 10% FBS, VitC, and TGF-β1 served as positive controls for the fibrosis model and treated gels were compared with them. The results showed that when the Rac1 pathway was blocked with NSC23766 inhibitor, SP was still able to induce contraction of the gels as much as SP alone (Fig. 5a). However, when the RhoA/ROCK pathway was blocked with Y-27632 inhibitor, SP was unable to induce contraction of the gels and the gel contraction remained on the level of negative control (corneal fibroblasts and 10% FBS only gels) (Fig. 5b), suggesting that SP induces pro-fibrotic changes through activation of the RhoA/ROCK pathway rather than the Rac1 pathway. We confirmed by western blot that SP increases the expression of RhoA and that this expression was diminished when the RhoA/ROCK pathway was blocked with Y-27632 inhibitor (Fig. 5c). To further study the role of the RhoA/ROCK pathway activation in SP-induced pro-fibrotic changes, we tested if this pathway is involved in FN gene expression. RT-qPCR showed that treatment of corneal fibroblasts with 10 μM Y-27632 together with 10^−5^M SP during the induction of fibrosis resulted in decreased FN gene expression when compared with SP only treated cells at both 2 days and 4 days after treatment (Fig. 5e). Additionally, we wanted to study if SP increases expression of the fibronectin EDA and, if so, if also that is controlled by activation of the RhoA/ROCK pathway. Immunofluorescence revealed that fibroblasts treated with 10^−5^M SP during the induction of fibrosis express more FN-EDA (green) than untreated control. When fibroblasts were pretreated with 10 μM Y-27632, and subsequently treated with 10^−5^M SP, the expression of FN-EDA decreased (Fig. 5f).

## Discussion

A normal corneal wound healing results in a healthy, transparent, and fully functional cornea. However, in some cases, the reparative process malfunctions, which leads to corneal haze, scarring, fibrosis, and even partial or full loss of vision. In our previous study [15], we focused on the role of SP in corneal wound healing. We showed that SP, which has been implicated in playing a role in wound healing of epithelial cells in various in vivo (rabbit and rat), as well as in vitro, models [32, 33, 39, 40] and which has been shown to be expressed by human keratocytes [27, 28], induced migration of keratocytes during the wound healing process and attracted neutrophils through secretion of interleukin-8 (IL-8) [15]. As induction of migration means that the normally quiescent keratocytes are activated and transition into fibroblasts and/or myofibroblasts, and as SP has been shown to be produced in the eye after induction of alkali burn in a rabbit eye model [32], we sought to study whether SP has an impact on other processes related to activation of keratocytes, such as excessive ECM production and expression of fibrotic markers, and whether it can play a role in the onset of corneal fibrosis in an in vitro human corneal fibrosis model.

First, we explored the effect of SP on the production of ECM proteins such as collagens I, III, and V, and lumican, and fibrotic markers α-SMA and fibronectin. SP treatment resulted in overproduction and excessive deposition of collagen I and V, which are phenomena that are hallmarks of fibrosis [5]. Enhanced expression of lumican further supports the pro-fibrotic role of SP, since lumican, which belongs to the small leucine-rich (SLRP) family of keratan sulfate proteoglycans, and is responsible for collagen fibril assembly and corneal transparency [41], also has been reported to regulate angiogenesis [42] and cell migration [43], and has been implicated in hepatic [44] and pulmonary fibrosis [45]. Collagen III, which under normal physiological conditions is expressed weakly in the cornea, but which expression increases greatly during pathological processes such as wound healing and inflammation [46], and is greatly enhanced in corneal scars [47], was also found to be overproduced by SP treatment, suggesting that SP might trigger fibrotic process in the cornea. Moreover, SP caused contraction of the collagen gels with embedded keratocytes, suggesting involvement and overexpression of α-SMA, an actin isoform typical of vascular smooth muscle cells [48], and a marker of myofibroblasts formation [4]. Another fibrotic marker enhanced by SP was fibronectin, a 440 kDa ECM glycoprotein which acts as a scaffold for binding collagens and other ECM components [49], which further promotes the fibrotic changes. Interestingly, we found that SP does not induce TGF-β1 production in keratocytes (data not shown). As TGF-β1 is the main factor responsible for activation of keratocytes and their transition into fibroblasts/myofibroblasts, it means that SP is able to conduct the transition of keratocytes into fibroblasts/myofibroblasts by another mechanism, perhaps by directly activating the RhoA/ROCK pathway. This process should be further studied.

The main scope of this study was to determine the role of SP during the onset of fibrosis in corneal fibroblasts by using an in vitro human corneal fibrosis model. In this model, VitC is used to induce synthesis and secretion of ECM, and TGF-β1 is used to stimulate overproduction and deposition of ECM, and transition of corneal fibroblasts into myofibroblasts. This model resulted in overproduction of ECM components (collagen I, collagen III, collagen V, and lumican), and expression of fibrotic markers fibronectin and α-SMA in newly formed contractile myofibroblasts (submitted data). In order to study the role of SP in the onset of fibrosis, SP was added to the experimental groups together with VitC and TGF-β1. As expected, in accordance with the results of SP on keratocytes, SP increased gene expression of collagen I, collagen III, collagen V, and lumican, further supporting the hypothesis that it promotes pathological changes in the cornea. Interestingly, secretion of collagen III and collagen V was not affected by SP treatment, perhaps suggesting an additional mechanism by which the overexpressed mRNA is deactivated by posttranscriptional modifications or that these collagens are not fully assembled by the cell. We also observed that SP increased contractile abilities of the corneal fibroblasts, therefore promoting their transition into myofibroblasts. The increased contraction correlated with increased levels of α-SMA in SP-treated cells. SP also increased gene expression and total fibronectin secretion in corneal fibroblasts. Taken together, our results strongly suggest that SP induces overproduction of ECM proteins and production of fibrotic markers, and induces transition of corneal fibroblasts into myofibroblasts with increased contractile abilities, therefore promoting the pathological state of fibrosis. In line with our findings concerning SP and keratocyte migration [15], the pro-fibrotic changes are induced by SP through activation of its preferred receptor NK-1R. We were able to show that when this receptor is blocked, SP cannot increase contractile abilities of the cells, and the levels of pro-collagen I, total fibronectin, and α-SMA expression remain at the level of fibrosis control cells (VitC and TGF-β1 treatment). Blocking of another SP receptor, NK-2R, did not affect the SP pro-fibrotic effect (data not shown). TGF-β induction of myofibroblast differentiation is driven by activation of Smad2/3/4 transcription factors; however, non-canonical pathways are also involved [12]. Based on our previous study [15], we were interested in determining whether SP-induced pro-fibrotic changes are triggered by activation of either RhoA/ROCK or Rac1 pathways. Activation of both of these pathways promotes transition of fibroblasts into myofibroblasts [50, 51] and has been implicated in promoting fibrosis [51, 52]. We used two inhibitors to study the pro-fibrotic SP mechanism: (1) NSC23766, a selective inhibitor of Rac1-GEF interaction, which prevents Rac1 activation by Rac-specific guanine nucleotide exchange factors (GEFs), and which inhibits Rac1-mediated cell functions. (2) Y-27632, a selective ROCK inhibitor. We discovered that inhibition of Rac1 did not affect SP actions. However, inhibition of the RhoA/ROCK pathway resulted in the inability of SP to induce contraction of corneal fibroblasts. Interestingly, also the experimental group in which fibrosis was induced together with the ROCK inhibitor alone did not show increased contractile abilities of the cells, suggesting that the induction of contractile forces and expression of α-SMA in corneal fibroblasts are generally driven by activation of the RhoA/ROCK pathway. To further confirm involvement of the RhoA/ROCK pathway in SP-induced pro-fibrotic changes, we investigated if its inhibition affects expression of fibrotic markers. Indeed, expression of α-SMA protein decreased when compared with SP only treated cells, and gene expression of fibronectin showed the same results. Fibronectin exists in two major forms: plasma fibronectin, a soluble secreted form, and cellular fibronectin, an insoluble form secreted by activated fibroblasts and myofibroblasts, containing the extra domain A (EDA) and extra domain B (EDB), products of alternative splicing [49, 53]. The plasma fibronectin is the most abundant form present in tissues under normal physiological conditions [54]. However, during wound healing or fibrosis, increased expression of EDA fibronectin (EDA-FN) has been reported [55]. Moreover, EDA-FN is essential for myofibroblasts differentiation, and it has been reported that in in vitro conditions, TGF-β1 increases levels of total fibronectin by promoting accumulation of the EDA-FN [56]. We established that SP increased expression of EDA-FN and that this was decreased when the RhoA/ROCK pathway was inhibited. Therefore, we suggest that SP-induced pro-fibrotic changes are driven by activation of the RhoA/ROCK pathway.

In conclusion, SP induces transition of quiescent keratocytes into a more contractile fibroblastic/myofibroblastic phenotype. This transition is marked by increased expression of ECM components such as collagens and lumican, and increased expression of fibrotic markers α-SMA and fibronectin. SP shows pro-fibrotic properties when administered at the onset of the fibrotic process in an in vitro human corneal fibrosis model. SP increases expression of collagens and lumican, as well as both α-SMA and fibronectin. This process is dependent on activation of the SP preferred receptor NK-1R, and involves activation of the RhoA/ROCK pathway. Taking into consideration our previous results on the role of SP in keratocyte migration, we can summarize that endogenous SP, released either by nerves or local cells [15] [28] during corneal injury and healing, promotes scarring and fibrosis of the cornea.

## Electronic supplementary material

ESM 1 (PDF 1402 kb)

ESM 2 (DOCX 3545 kb)
