# Supplementary material for: Substance P induces fibrotic changes through activation of the RhoA/ROCK pathway in an in vitro human corneal fibrosis model
Source: J Mol Med (Berl). 2019 Aug 9;97(10):1477–89. doi: 10.1007/s00109-019-01827-4 (PMC6746877; doi:10.1007/s00109-019-01827-4)

Full unedited gels for Figure 3C.

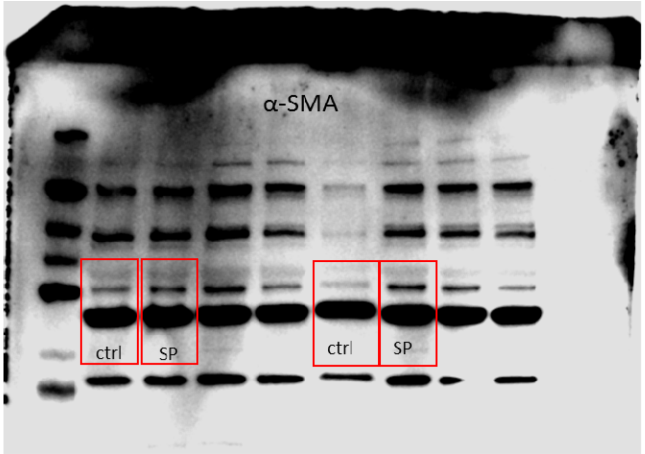

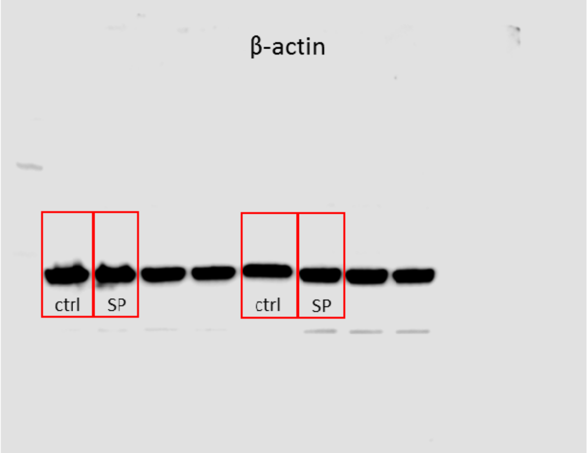

Full unedited gels for Figure 4D.

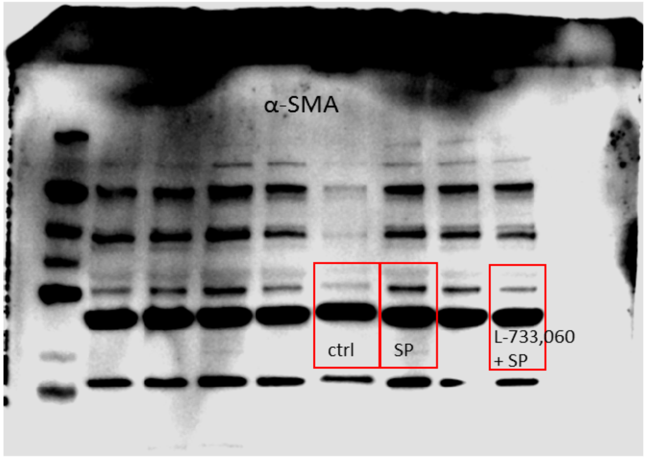

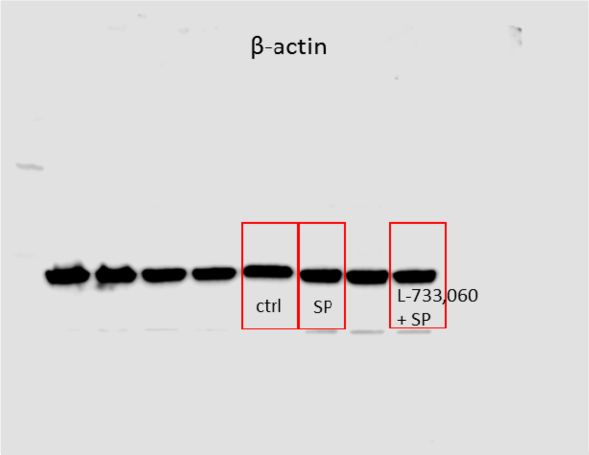

Full unedited gels for Figure 5C.

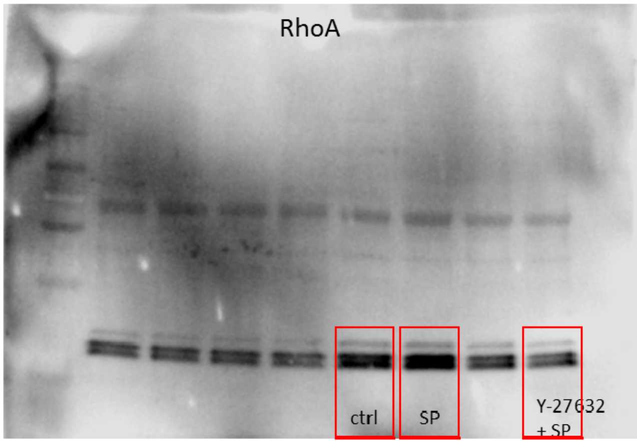

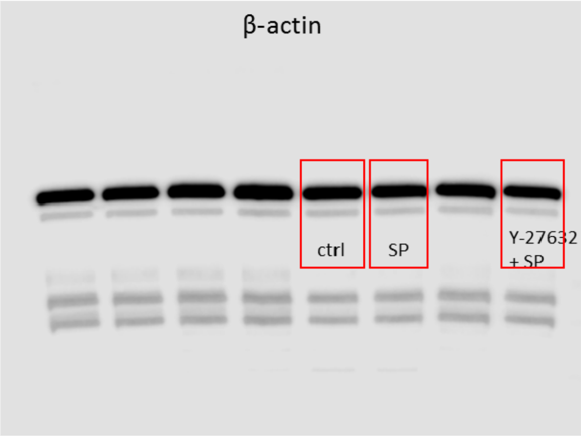

Full unedited gels for Figure 5D.

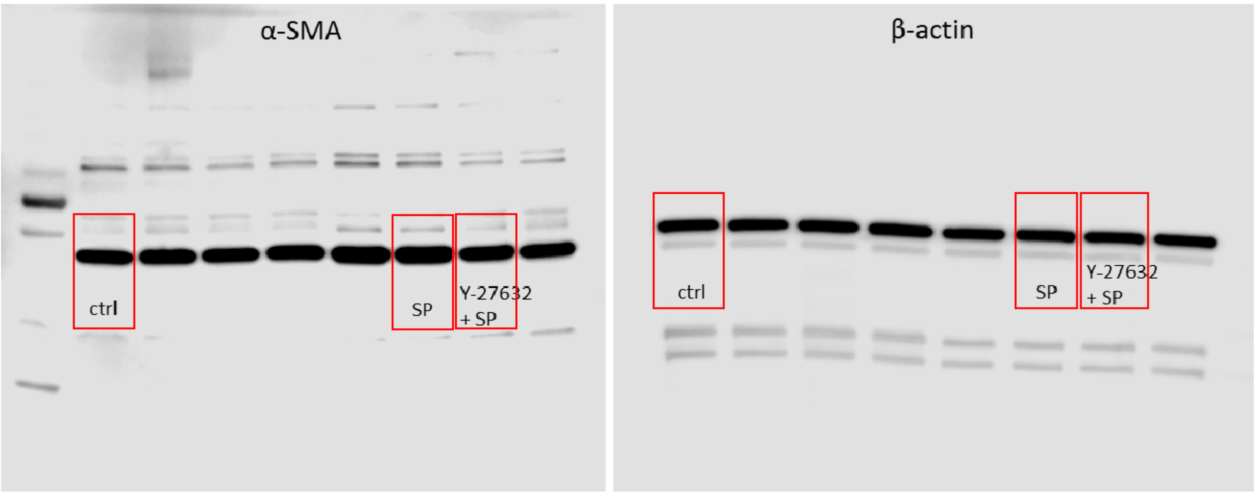

Supplement: Supplementary file 1 — (PDF 1402 kb) [file 109_2019_1827_MOESM1_ESM.pdf]
