# Supplementary material for: Substance P induces fibrotic changes through activation of the RhoA/ROCK pathway in an in vitro human corneal fibrosis model
Source: J Mol Med (Berl). 2019 Aug 9;97(10):1477–89. doi: 10.1007/s00109-019-01827-4 (PMC6746877; doi:10.1007/s00109-019-01827-4)
Supplement: Supplementary file 2 — (DOCX 3545 kb) [file 109_2019_1827_MOESM2_ESM.docx]

**SUPPLEMENTAL FIGURE 1**


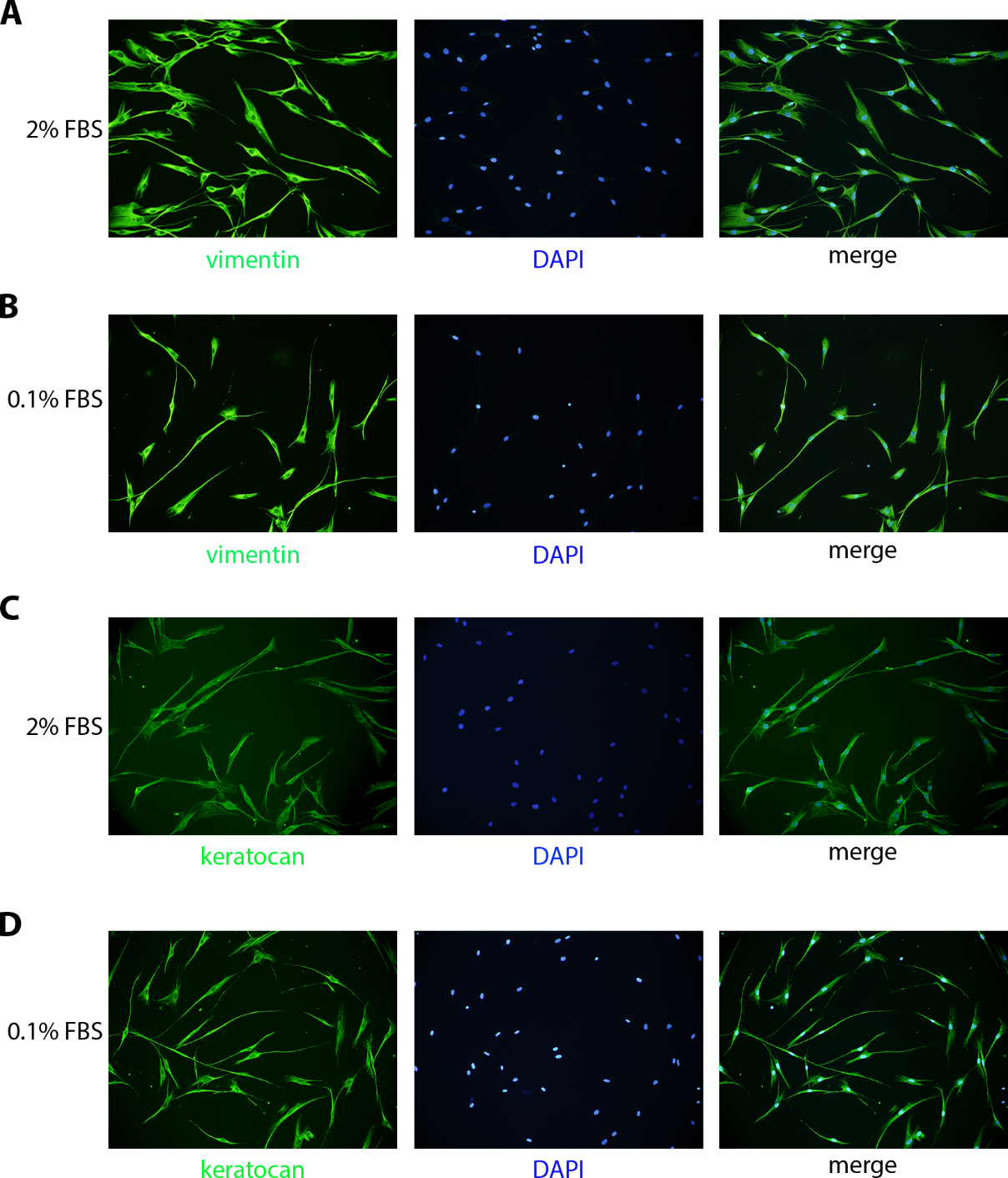


**Supplemental Figure 1. Morphology and expression of keratocyte marker in keratocytes grown in DMEM/F-12 2% FBS and DMEM/F-12 0.1%.** (A) Morphology of keratocytes grown in DMEM/F-12 2% FBS, as revealed by vimentin staining (green), shows the spindle-like, elongated shape of the cells. (B) Morphology of keratocytes grown in DMEM/F-12 0.1% FBS as revealed by vimentin staining (green), shows the spindle-like, elongated shape of the cells. (C) Keratocytes grown in DMEM/F-12 2% FBS express keratocan (green). (D) Keratocytes grown in DMEM/F-12 0.1% FBS express keratocan (green).

**SUPPLEMENTAL FIGURE 2**

**
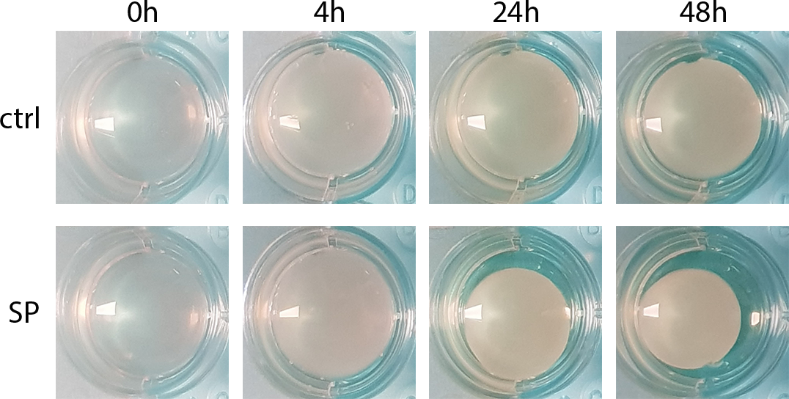
**

**Supplemental Figure 2. Representative images of contraction assays of keratocytes treated with SP.** Ctrl – represents DMEM/F-12 0.1% FBS control. SP – represents DMEM/F-12 0.1% FBS + SP.

**SUPPLEMENTAL FIGURE 3**


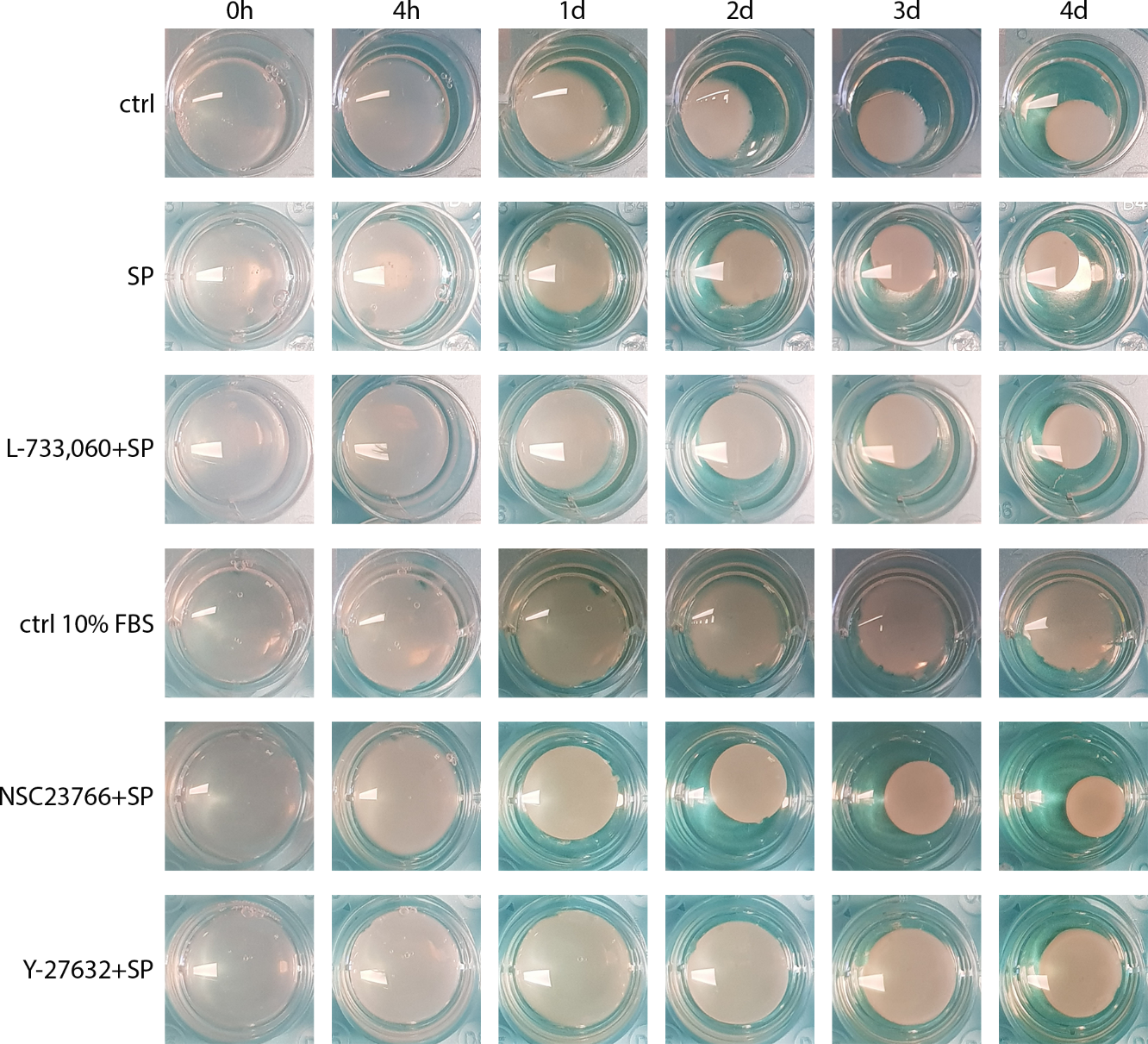


**Supplemental Figure 3. Representative images of contraction assays.** Ctrl – represents DMEM/F-12 10% FBS + VitC + TGFβ1 control. Ctrl 10% FBS – represents DMEM/F-12 10% FBS only control.
